# Prognosis of critically ill patients with cirrhosis and acute kidney injury initiated on dialysis

**DOI:** 10.1186/s12882-026-05096-5

**Published:** 2026-06-11

**Authors:** Eric Kerns, Ahmed Agameya, Ahmed Abdelfattah, Janine Molino, Sandipan Shringi, Avignat Patel

**Affiliations:** 1 Division of Kidney Disease and Hypertension, Department of Medicine, Warren Alpert Medical School and Brown University Health, Providence, RI USA; 2 Division of Pulmonary and Critical Care Medicine, Department of Medicine, Lahey Hospital and Medical Center, Burlington, MA USA; 3 Division of Gastroenterology, Department of Medicine, Lahey Hospital and Medical Center, Burlington, MA USA; 4 Lifespan Biostatistics Core, Rhode Island Hospital and Department of Orthopedics, Warren Alpert Medical School, Brown University, Providence, RI USA; 5 Division of Pulmonary and Critical Care Medicine, Department of Medicine, Lahey Hospital and Medical Center and Tufts University School of Medicine, Burlington, MA USA

**Keywords:** Acute kidney injury, Cirrhosis, Intensive care unit, Critical care, Kidney replacement therapy

## Abstract

**Background:**

Acute kidney injury (AKI) is a common complication in critically ill patients with decompensated cirrhosis who often require kidney replacement therapy (KRT) but prognostic uncertainty remains in critically ill ICU patients, particularly regarding short- and medium- term outcomes after KRT initiation.

**Methods:**

We conducted a retrospective, single center cohort analysis of critically ill patients with cirrhosis and AKI requiring KRT in the medical ICU of a tertiary care liver transplant center. We gathered data pertaining to CLIF-C ACLF and MELD-Na on the day of ICU admission. Survival outcomes of interest included days to death from the start of KRT, and at one- and six-month interval.

**Results:**

131 patients met the inclusion criteria of which 21 were listed for liver transplantation at time of ICU admission. 86.3% of patients were diagnosed with acute tubular necrosis (ATN) and the remaining with HRS-AKI. 69.5% of patients were prescribed Continuous Veno Venous Hemofiltration (CVVH) as initial KRT modality. Mean clinical severity scores were MELD-Na 33.1 (SD 8.3) and CLIF-C ACLF score 63.4 (SD 10.3). 21.4% and 15.3% of the 131 patients included, were alive at 1 and 6 months after ICU admission. Median survival time from KRT initiation in ICU was 5 days (IQR 3-12.5 days). Survival did not differ significantly by AKI etiology or transplant listing status although this is limited by small sample size and dynamic nature of transplant listing status. In unadjusted analyses, survival differed by initial KRT modality, with higher mortality among patients initiated on CVVH; however, in multivariable Cox regression, only CLIF-C ACLF score [HR 1.03 (1.00- 1.05), *p* = 0.04], platelets [HR 0.997 (0.994–0.999), *p* = 0.02] and INR [HR 1.22 (1.00-1.49), *p* = 0.05] were significantly associated with survival time.

**Conclusions:**

AKI requiring KRT in critically ill patients with cirrhosis is associated with limited survival. Mortality was primarily associated with overall severity of acute-on-chronic liver failure, as reflected by CLIF-C ACLF score, rather than MELD-Na. No clear differences were observed by AKI etiology or transplant listing status. Although unadjusted survival differed by initial KRT modality, this likely reflects confounding by illness severity rather than a modality-specific effect.

**Clinical trial number:**

Not applicable.

## Introduction

Cirrhosis accounts for approximately 49,000 deaths per year in the United States [1]. Acute kidney injury (AKI) represents the fifth leading cause of hospitalization and the third leading cause of intensive care unit (ICU) admission in this population [2] and associates with medical and hepatic complications, hospital length of stay, and mortality in a stage-dependent manner [3–5]. It also adds to the cost burden in the care of these patients [6]. AKI is a common complication of cirrhosis, occurring in 20 to 50% of hospitalized patients with ascites [6–9]. Although AKI in this patient population can be due to pre-renal, renal or post renal causes, the most common etiologies include hypovolemia, acute tubular necrosis (ATN), a prerenal, vasoconstriction state unique to cirrhosis called hepatorenal syndrome-AKI (HRS-AKI), and a functional indolent decline in kidney function called HRS-non-AKI [7, 10, 11].

Distinction between these conditions can be challenging and while emerging biomarkers such as neutrophil gelatinase-associated lipocalin (NGAL) could prove beneficial in diagnosing ATN, they are not widely available. However, due to differences in management and prognosis, it is important to diagnose them accurately. About 50% cases of AKI in hospitalized patients with cirrhosis have a prerenal azotemia and improve with volume resuscitation, 30% have ATN which can be expected to improve with supportive care and 20% have HRS-AKI which require prompt treatment [11–14]. Differentiating these conditions is essential, particularly in cases where liver transplantation is not an option, for goals of care planning and by extension, for making decisions regarding the use of dialysis [15, 16] which is often needed as a bridge to hepatic recovery or to transplantation.

While the indications for dialysis or kidney replacement therapy (KRT) for AKI in patients with cirrhosis are similar to those in the general population, these patients experience numerous dialysis related complications specific to liver failure. These include bleeding, infection, encephalopathy, and systemic hypotension, all of which may necessitate hospital-level care and in the most severe cases, ICU transfer for continuous KRT [9, 13, 17].

The number of patients with decompensated cirrhosis and AKI receiving KRT in the US is increasing [18] and yet there are only a few large studies investigating survival outcomes in these patients – particularly in the non-transplant population. The available studies generally agree that transplant-free survival for hospitalized patients is on the order of days to weeks once they get sick enough to necessitate initiation of KRT [19–21]. Only one study explored whether the cause of AKI and transplant-listing status affected survival [22] while only one study focused exclusively on critically ill patients with cirrhosis and found that 92% of patients who continued to require KRT after ICU died within the year [23]. While this study used KRT for standard indications, it did not differentiate between the causes of AKI. In addition, the initiation of KRT could be influenced by transplant listing status with transplant ineligible patients having poor survival to those listed. However, a study found that 1 out of 6 transplant ineligible patients was still alive at 6 months highlighting the uncertainty regarding their prognosis [24]. Liver based prognostic models such as Model for End-Stage Liver Disease (MELD-Na) do not address the multiorgan dysfunction which may define outcomes in critically ill patients where Acute on Chronic Liver Failure (ACLF), characterized by acute hepatic decompensation, systemic inflammation and failure of one or more organs, results in higher mortality. In contrast, Chronic Liver Failure-Consortium Acute on Chronic Liver Failure (CLIF-C ACLF) score integrates both hepatic and extrahepatic parameters making it useful for the critically ill patients with kidney dysfunction [25].

We therefore performed a retrospective cohort analysis of critically ill patients with cirrhosis and AKI requiring KRT initiation in the medical ICU of a tertiary care liver transplant center. The purpose of this study was to determine short- and medium-term survival in this population and to identify prognostic factors associated with mortality among critically ill patients with cirrhosis and AKI initiated on KRT including whether outcomes differed by AKI etiology (ATN vs. HRS-AKI), transplant listing status, and overall severity of acute-on-chronic liver failure.

## Methods

### Patient population and data collection

We performed a retrospective cohort study of all ICU patients with cirrhosis and AKI requiring KRT for either HRS-AKI or ATN between March of 2015 and December of 2018 at a large liver transplant center (Lahey Hospital and Medical Center, Burlington, Massachusetts). This hospital serves as a regional referral center for patients with end-stage liver disease and performs approximately 70–100 liver transplantations each year.

The department of medical informatics generated a list of patients with a past medical history or a diagnosis of cirrhosis who were admitted to the medical ICU. The diagnosis filter used to identify patients with cirrhosis was based on a Systematized Nomenclature of Medicine – Clinical Terms (SNOMED – CT) grouper. A patient database had been created for a larger study examining the outcomes and survival of patients with cirrhosis admitted to the medical ICU, and patients who underwent KRT during the relevant ICU admission were included. Subjects were then excluded if they had CKD G5D or acute hepatitis.

Patients were treated primarily by the medical ICU team and initiated dialysis at the discretion of the consulting nephrologist according to standard indications for KRT, including volume overload, hyperkalemia, metabolic acidosis, and uremia refractory to medical management. Patients who required pressors for hemodynamic support at the time of dialysis initiation or who were considered hemodynamically unstable by the consulting nephrologists, were started on continuous venovenous hemofiltration (CVVH) whereas those who were hemodynamically stable started with conventional hemodialysis (HD). The decision to initiate KRT or the modality used was not influenced by transplant status and all patients were on KRT prior to transplantation. KRT was continued post transplantation at the discretion of consulting nephrologist. In all cases, dialysis was initiated via a temporary or tunneled dialysis catheter. HD treatments were typically prescribed 3 days per week, for 3 h each session, with a blood flow rate of 400 mL/min and a dialysate flow rate of 800 mL/min. CVVH was typically prescribed with a blood flow rate of 200 mL/min to achieve an effluent volume of 30 mL/kg/hr. Peritoneal dialysis was not offered as a modality.

All potential subjects underwent review of the medical record to determine accuracy of the diagnostic codes, medical history, and reasons for ICU admission. Data were gathered independently by one of the investigators (EK) and reviewed for any discrepancies by two of the other investigators (AP and AA). MELD-Na score and the CLIF-C ACLF score were calculated on the day of ICU admission. Dynamic measures of illness severity during the ICU course, including timing of KRT initiation relative to ICU admission and granular hemodynamic parameters, were not recorded. The baseline labs were collected on the day of dialysis initiation. Liver transplant status was determined from the notes of the consulting hepatologist at the time of ICU admission.

### Definitions

Definitions and causes of cirrhosis were made based on notes of the consulting hepatologist and causes of ICU admission were based on the medical ICU team documentation. Notes of the consulting nephrologist on the date of initial consultation were reviewed in detail. In cases of pre-existing CKD, a patient was determined to have end-stage renal disease or CKD G5D if he or she was dialysis-dependent for greater than 3 months prior to the ICU admission. Patients with CKD were identified by review of the past medical history, problem lists, and notes of the consulting nephrologist. Similarly, medical comorbidities including coronary artery disease, hypertension, and diabetes were determined by review of the medical history and problem lists on the date of ICU or hospital admission.

Causes of AKI were determined by chart review, using notes of the nephrologist on the day of initial consultation, which generally included examination of the urine sediment by microscopy. AKI etiology was initially abstracted by one investigator (E.K.) and subsequently reviewed independently by two additional investigators (A.P. and A.A.) for concordance. Any discrepancies were resolved by consensus review of the medical record. Formal inter-observer agreement statistics were not assessed because AKI etiology was determined by consensus adjudication, and individual reviewer classifications were not retained for independent agreement analysis. ATN was considered to be the cause of AKI when the nephrologist directly referenced ATN in the assessment, when there was documentation of muddy brown casts on urine sediment analysis, or when the clinical history supported ischemic or nephrotoxic AKI as from shock or sepsis. HRS-AKI was diagnosed after other causes of AKI were excluded and based on the assessment by the consulting nephrologist. Urine sodium, when available at or around the onset of AKI, was recorded, but this was not used in the determination of cause of AKI. In cases of mixed type HRS-AKI and ATN, the cause of AKI was considered to be ATN given that HRS-AKI is a diagnosis of exclusion. This method of diagnosis was consistent with a previous study [22]. Recovery from AKI was defined as recovery of sufficient kidney function to stop dialysis treatments either during the hospitalization or after hospital discharge, based on review of the medical record.

### Outcomes

The primary outcomes of interest included days to death from start of KRT, one-month survival, and six-month survival. Subjects were also assessed for dialysis-dependence at one month and six months from the start of KRT. Additional outcomes of interest included the occurrence of liver transplantation, simultaneous liver-kidney (SLK) transplantation, or kidney transplantation, and consultation by palliative care medicine service during the admission and the code status at time of death. Time-to-event analyses were anchored at the date of initiation of KRT. Patients who were alive at the end of follow-up were censored at their last known follow-up date. Patients who underwent liver, simultaneous liver–kidney, or kidney transplantation during follow-up were censored at the time of transplantation. Transplantation was not treated as a mortality event.

### Statistical analysis

Data was imported into SAS version 9.4 (SAS Institute Inc., Cary, NC) for data management and analysis. Descriptive statistics were obtained for the patient characteristics and study outcomes. Mean and standard deviation were reported for the normally distributed continuous variables while median and interquartile range were reported for the non-normally distributed continuous variables. Frequency and percentage were reported for the binary and categorical variables. Chi-square tests were used to compare gender, comorbid conditions, primary reason for ICU admission, characteristics on day of ICU admission, etiology of AKI, and initial KRT modality by vital status 1 and 6 months after ICU admission. Independent samples t tests were used to compare patient age by vital status 1 and 6 months after ICU admission. Kaplan-Meier curves and Cox proportional hazards regression models were estimated to examine the effect of patient characteristics on overall survival. Given the limited number of survival events and the exploratory nature of this analysis, multivariable (adjusted) Cox proportional hazards models were constructed using a parsimonious variable selection strategy to reduce the risk of overfitting. Variables demonstrating a statistically significant association with survival (*p* < 0.05) in univariable (unadjusted) Cox regression analyses were considered for inclusion in the multivariable model. Proportional hazards assumptions were not formally tested. Analyses were performed using a complete-case approach, such that patients with missing values for variables included in a given analysis were excluded from that specific analysis. The extent of missing data was not formally assessed. However, given that the multivariable model included 111 death events from a cohort of 131 patients, missingness was likely modest. No formal sensitivity or secondary analyses were performed given the limited sample size and number of outcome events to avoid further loss of statistical power. Additionally, with limited number of events relative to candidate variables, potential for residual confounding remains. Hazard ratios, along with their 95% confidence intervals, were reported. *P* < 0.05 was used to determine statistical significance.

### Ethics statement

The Lahey Hospital and Medical Center Institutional Review Board approved this study (IRB# 1097668). The need for informed consent was waived.

## Results

### General demographics

**Fig. 1:**
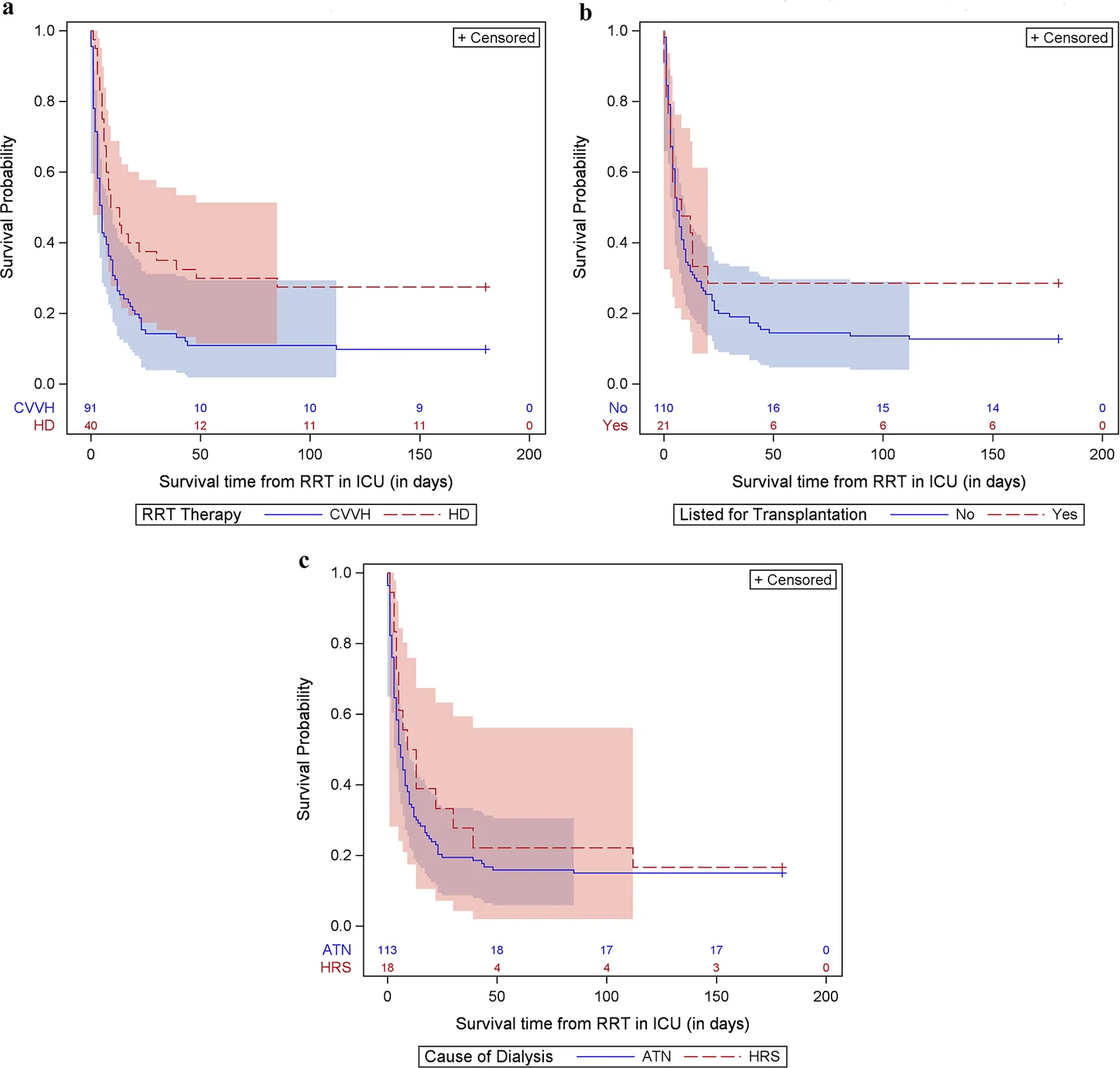
**a**. Unadjusted survival by initial KRT therapy (log-rank *p* = 0.002, descriptive analysis only, not adjusted for illness severity). Survival probability from initiation of KRT in the ICU stratified by initial KRT modality: continuous venovenous hemofiltration (CVVH; blue solid line, *n* = 91) versus intermittent hemodialysis (HD; red dashed line, *n* = 40). Shaded areas represent 95% confidence intervals. Numbers at risk are shown below the x-axis at each time interval. Censored observations are indicated by (+) symbols. **b**. Survival by listed for transplantation (*p* = 0.28). Survival probability from initiation of KRT in the ICU stratified by transplant listing status: not listed (blue solid line, *n* = 110) versus listed for transplantation (red dashed line, *n* = 21). Shaded areas represent 95% confidence intervals. Numbers at risk are shown below the x-axis at each time interval. Censored observations are indicated by (+) symbols. **c**. Survival by cause of AKI (*p* = 0.35). Survival probability from initiation of KRT in the ICU stratified by cause of AKI: acute tubular necrosis (ATN; blue solid line, *n* = 113) versus hepatorenal syndrome (HRS; red dashed line, *n* = 18). Shaded areas represent 95% confidence intervals. Numbers at risk are shown below the x-axis at each time interval. Censored observations are indicated by (+) symbols

All patients with cirrhosis admitted to the medical ICU during the study period were identified using diagnostic codes. From this cohort, patients who required initiation of KRT during the ICU admission were included for further review. This cohort included only patients in whom KRT was initiated; the total number of ICU patients with cirrhosis and AKI who were not offered KRT could not be reliably ascertained. Accordingly, the study cohort reflects a selected population in whom KRT was initiated, rather than all patients with cirrhosis and AKI in the ICU. Patients were excluded if they had CKD G5D or acute hepatitis. After application of inclusion and exclusion criteria, 131 patients comprised the final analytic cohort (Table 1). Primary analyses focused on overall survival and prognostic factors associated with mortality. Additional findings related to kidney recovery, transplantation, and subgroup comparisons are reported as secondary, descriptive analyses. The mean age was 56.3 years (SD 10) and 58% were male. The most common primary reason for ICU admission was shock (27.5%) with AKI accounting for 7.6% of primary ICU diagnoses. The majority of subjects had alcoholic cirrhosis (61.8%) with most having decompensated cirrhosis: ascites (93.1%), encephalopathy (42.8%) and gastrointestinal bleeding (40.5%). A significant number of subjects required advanced critical care support with mechanical ventilation (64.9%) and/or IV vasopressors (68.7%). 21 of the 131 subjects were listed for liver transplant at time of ICU admission. 86.3% in the cohort were diagnosed with ATN as etiology of AKI versus 13.7% with HRS-AKI. 69.5% of subjects were prescribed CVVH as initial KRT modality. Clinical severity scores were calculated: mean MELD-Na 33.1 (SD 8.3) and mean CLIF-C ACLF score 63.4 (SD 10.3). 21.4% of subjects were alive at 1 month after ICU admission and 15.3% alive at 6 months after ICU admission (observed survival proportions anchored at ICU admission). Median survival time from KRT initiation in ICU was 5 days (IQR 3-12.5). Time-to-event analyses, including Kaplan–Meier estimates and Cox regression, were anchored at the date of KRT initiation (Fig. 1).

**Table 1:** Patient characteristics

Characteristic	Mean (SD) or *N* (%)
Age – years	56.3 (10)
Male gender	76 (58%)
Reason for ICU Admission	
* Shock*	*36 (27.5%)*
* Gastrointestinal bleed*	*22 (16.8%)*
* Altered mental status*	*21 (16.0%)*
* Respiratory failure*	*20 (15.3%)*
* Sepsis*	*19 (14.5%)*
* AKI*	*10 (7.6%)*
* Other*	*3 (2.3%)*
Critical care support	
* Mechanically ventilated*	*85 (64.9%)*
* Intravenous vasopressor use*	*90 (68.7%)*
Listed for Liver Transplantation	21 (16%)
Number Transplanted (total)	9
* Liver*	*6*
* SLK*	*2*
* Kidney*	*1*
Cirrhosis Etiology	
* EtOH*	*81 (61.8%)*
* Hepatitis C*	*20 (15.3%)*
* Non-alcoholic steatohepatitis*	*22 (16.8%)*
* Autoimmune hepatitis*	*3 (2.3%)*
* Cryptogenic*	*4 (3.1%)*
* Hepatitis B*	*3 (2.3%)*
* Other*	*14 (10.7%)*
Cirrhosis Complications	
* Ascites*	*122 (93.1%)*
* Encephalopathy*	*56 (42.8%)*
* Gastrointestinal bleeding*	*53 (40.5%)*
* Spontaneous bacterial peritonitis*	*22 (16.8%)*
* Hepatocellular cancer*	*9 (6.9%)*
Clinical Severity Scores	
* MELD-Na*	*33.1 (8.3)*
* CLIF-C ACLF Score*	*63.4 (10.3)*
Cause of AKI	
* Acute tubular necrosis*	*113 (86.3%)*
* Hepatorenal syndrome*	*18 (13.7%)*
Initial KRT Modality	
* Intermittent hemodialysis*	*40 (30.5%)*
* Continuous venovenous hemofiltration*	*91 (69.5%)*
Labs on KRT Initiation	
* WBC*	*15.7 (8.7)*
* Na*	*135 (12.9)*
* Creatinine*	*4.2 (1.9)*
* Albumin*	*2.9 (0.96)*
* Total bilirubin*	*13.5 (12.8)*
* INR*	*2.6 (1.14)*
Alive at 1 month after ICU admission	28 (21.4)
Alive at 6 months after ICU admission	20 (15.3)
Days to Death from KRT initiation in ICU, median (IQR)	5 (3, 12.5)

Values are presented as mean (SD) for continuous variables and number (%) for categorical variables. Reason for ICU admission reflects the primary admission diagnosis as documented by the medical ICU team

Abbreviations: AKI, acute kidney injury; SLK, simultaneous liver and kidney transplant; EtOH, alcohol; KRT, kidney replacement therapy; WBC, White blood cell count; Na, serum sodium; INR, International Normalized Ratio

### One-month survival

21.4% (28 of 131) of all subjects were alive at 1 month (observed proportions from ICU admission). Table 2 summarizes demographics and characteristics of all subjects by vital status at 1 month. Gender, reason for ICU admission, comorbidities, and etiology of AKI were similar between those alive and dead at 1 month. Those alive at 1 month were younger with a mean age of 50.7 years (9.55) vs 57.8 years (9.98), less likely to be on mechanical ventilation: 14 (50%) vs 71 (68.9%), and less likely to have received IV vasopressors: 12 (42.9%) vs 78 (75.7%). Those alive at 1 month were more commonly initiated on HD compared with CVVH: 13 (46.4%) vs 78 (75.7%). They also had a lower mean CLIF-C ACLF score: 56.0 (8.2) vs 65.4 (9.9) and MELD-Na score: 30.2 (9.9) vs 33.9 (7.7).

**Table 2:** Vital Status at one month and six months from ICU admission (Observed survival proportions)

	Vital Status at 1 Month (*n* = 28)			Vital Status at 6 Months (*n* = 20)		
	Dead Mean (SD) or *N* (%)	Alive Mean (SD) or *N* (%)	*p*	Dead Mean (SD) or *N* (%)	Alive Mean (SD) or *N* (%)	*p*
Age	57.8 (10.0)	50.7 (9.6)	0.001	57.5 (10.1)	49.8 (8.8)	0.002
Gender						
Male Female	63 (61.2%) 40 (38.8%)	13 (46.4%) 15 (53.6%)	0.16	66 (59.5%) 45 (40.5%)	10 (50.0%) 10 (50.0%)	0.43
Comorbid Conditions						
Diabetes mellitus Coronary artery disease Chronic kidney disease Hypertension	28 (27.2%) 13 (12.6%) 25 (24.3%) 54 (52.4%)	6 (21.4%) 1 (3.6%) 6 (21.4%) 11 (39.3%)	0.54 0.30 0.75 0.22	31 (27.9%) 14 (12.6%) 27 (24.3%) 57 (51.4%)	3 (15.0%) 0 (0.0%) 4 (20.0%) 8 (40.0%)	0.28 0.13 0.78 0.35
Primary Reason for ICU Admission						
Gastrointestinal bleed Sepsis Shock Altered mental status Respiratory failure Renal failure Other	19 (18.5%) 16 (15.5%) 30 (29.1%) 13 (12.6%) 17 (16.5%) 7 (6.8%) 1 (1.0%)	3 (10.7%) 3 (10.7%) 6 (21.4%) 8 (28.6%) 3 (10.7%) 3 (10.7%) 2 (7.1%)	0.16	20 (18.0%) 16 (14.4%) 32 (28.8%) 15 (13.5%) 17 (15.3%) 8 (7.2%) 3 (2.7%)	2 (10.0%) 3 (15.0%) 4 (20.0%) 6 (30.0%) 3 (15.0%) 2 (10.0%) 0 (0.0%)	0.63
Characteristics on day of ICU Admission						
Mechanically ventilated Intravenous vasopressor use CLIF-C ACLF score MELD-Na score Listed for liver transplantation	71 (68.9%) 78 (75.7%) 65.4 (9.9) 33.9 (7.7) 15 (14.6%)	14 (50%) 12 (42.9%) 56.0 (8.2) 30.2 (9.9) 6 (21.4%)	0.06 0.001 < 0.0001 0.03 0.38	74 (66.7%) 81 (73.0%) 64.5 (10.2) 33.3 (8.5) 15 (13.5%)	11 (55.0%) 9 (45.0%) 57.2 (8.9) 32.4 (7.1) 6 (30.0%)	0.31 0.01 0.003 0.65 0.06
Etiology of AKI						
Acute tubular necrosis Hepatorenal syndrome	91 (88.4%) 12 (11.6%)	22 (78.6%) 6 (21.4%)	0.36	96 (86.5%) 15 (13.5%)	17 (85.0%) 3 (15.0%)	0.99
Initial KRT Modality						
Intermittent hemodialysis Continuous venovenous hemofiltration	25 (24.3%) 78 (75.7%)	15 (53.6%) 13 (46.4%)	0.003	29 (26.1%) 82 (73.9%)	11 (55.0%) 9 (45.0%)	0.01

Values are presented as mean (SD) for continuous variables and number (%) for categorical variables. P-values compare alive versus dead groups at each time point. Continuous variables were compared using independent samples t tests, and categorical variables were compared using chi-square tests. Survival status reflects observed survival proportions from ICU admission

### Six-month survival

15.3% (20 of 131) of all subjects were alive at 6 months (observed proportions from ICU admission). Table 2 summarizes demographics and characteristics of all subjects by vital status at 6 months. Gender, reason for ICU admission, comorbidities and etiology of AKI were again similar between those subjects alive and dead at 6 months. Those alive were younger with a mean age of 49.8 years (8.8) vs 57.5 years (10.1), less likely to have received vasopressors: 9 (45%) vs 81 (73%). Those alive at 6 months were more commonly initiated on HD compared with CVVH: 9 (45%) vs 82 (73.9%). They also had a lower mean CLIF-C ACLF score: 57.2 (8.9) vs 64.5 (10.2) but a similar MELD-Na. Of the 20 subjects alive at 6 months, 6 had been transplanted (4 liver, 2 SLK) and 2 were transplanted after 6 months (1 liver, 1 kidney).

### Regression analysis for survival time

We performed a Cox regression analysis for survival time from KRT initiation in the ICU using the clinical variables shown in Table 2. Variables that reached significance (*p* < 0.05) in the unadjusted regression analysis were included in the adjusted analysis (Table 3). Given the limited number of events and the exploratory modeling strategy, these adjusted estimates should be interpreted cautiously. In the multivariable analysis, CLIF-C ACLF score was associated with survival time [HR 1.03 (1.00–1.05), *p *= 0.04]. Platelet count [HR 0.997 (0.994–0.999), *p *= 0.02] and INR [HR 1.22 (1.00–1.49), *p *= 0.05] were also significant. However, these findings should be interpreted cautiously given the exploratory modeling approach and potential for residual confounding.

**Table 3:** Cox regression analysis for survival time (adjusted)

Characteristic	HR (95% CI)	*p*
Age	1.01 (0.99, 1.04)	0.30
IV vasopressor use	1.52 (0.92, 2.51)	0.11
CLIF C-ACLF score	1.03 (1.00, 1.05)	0.04
Ascites	0.80 (0.29, 2.21)	0.67
CAD	1.98 (0.98, 3.98)	0.06
Platelets	0.997 (0.994, 0.999)	0.02
INR	1.22 (1.00, 1.49)	0.05
AST	1.00 (1.00, 1.00)	0.57
ALT	1.00 (1.00, 1.00)	0.22
Initial KRT		
CVVH	REF	
HD	0.74 (0.46, 1.19)	0.22

Abbreviations: IV, Intravenous; CAD, Coronary artery disease; INR, International Normalized Ratio; AST, Aspartate aminotransferase; ALT, Alanine aminotransferase; KRT, Kidney replacement therapy; CVVH, continuous venovenous hemofiltration; HD, hemodialysis

Lastly, we performed survival probability estimates based on initial KRT modality (HD vs. CVVH), transplant listing status and etiology of AKI (HRS-AKI vs. ATN) (Fig. 1) using the Kaplan-Meier method. A multivariable Cox proportional hazards model for survival time from KRT initiation was constructed and included 111 death events. In unadjusted Kaplan–Meier analyses, survival probability differed by initial KRT modality (log-rank p = 0.002). However, this association should be interpreted cautiously, as patients initiated on CVVH were more likely to have greater illness severity. In multivariable Cox regression analysis adjusting for severity and clinical covariates, initial KRT modality was not independently associated with survival.

### Kidney recovery and transplantation

As a secondary descriptive analysis, we examined kidney recovery and transplantation outcomes. 9.2% (12 of 131) of all subjects recovered kidney function, 10 of whom had AKI secondary to ATN and 2 with AKI secondary to HRS-AKI. Approximately half of the patients who recovered kidney function did so by 1 month, and the other half by 6 months. All but 1 of the patients who recovered kidney function were alive at 6 months. 6.9% (9 of 131) of patients underwent organ transplantation (6 liver, 2 SLK, and 1 kidney), of whom 88.9% (8 of 9) were alive at 6 months. Of the patients who underwent liver transplantation and were alive at 6 months, 75% (6 of 8) recovered kidney function. These findings are limited by the small number of listed patients and the dynamic nature of transplant listing status.

### Palliative care involvement and end-of-life care

Palliative care consultation occurred in approximately one-quarter of patients during the hospitalization. Among patients who died during follow-up, 96% were receiving hospice or comfort-measures-only care at the time of death. Cardiopulmonary resuscitation (CPR) prior to death was uncommon, occurring in only three patients in the cohort. Palliative care involvement differed by transplant listing status, with consultations occurring more frequently among patients who were not listed for liver transplantation (30%) compared with those who were listed at the time of ICU admission (5%).

## Discussion

AKI requiring KRT in decompensated cirrhosis carries a grave prognosis with significant morbidity and mortality. Whether KRT should be offered to this patient population remains controversial, particularly when liver transplantation is not an option due to social (i.e., active substance abuse) or medical (i.e., multi-organ failure) contraindications. There is a lack of data to guide goals of care discussions and medical decision-making, particularly in critically ill patients. Thus, we conducted this retrospective, single-center observational study to describe short- and medium-term outcomes among critically ill patients with cirrhosis and AKI requiring KRT and to identify clinical factors associated with mortality. We confirmed a high overall mortality with median survival measurable in days. Overall illness severity, as reflected by CLIF-C ACLF score, was associated with survival, whereas initial KRT modality was not. Although unadjusted analyses suggested differences in survival by initial KRT modality, this finding is most consistent with confounding by indication, as patients receiving continuous therapies were more likely to be hemodynamically unstable. Accordingly, these results should be interpreted as hypothesis generating rather than indicative of a modality-specific effect. Importantly, survival in this cohort should be interpreted as conditional on selection for KRT initiation, rather than as an unbiased estimate of prognosis for all ICU patients with cirrhosis and AKI. We also found no clear association between etiology of AKI or transplant listing status and survival.

Previous studies of KRT in the cirrhotic patient population have been limited by relatively small sample sizes. A case series of four patients with HRS-AKI undergoing HD found that while dialysis prolonged survival, patients spent on average one-third of their survival days in the hospital [17]. Parsons and Wilkinson described 0% survival in 25 hospitalized patients with cirrhosis initiating HD and peritoneal dialysis [20]. Similarly, in a group of 102 liver transplant candidates who started dialysis for AKI, Wong found a median transplant-free survival of 7 days with overall survival to transplantation of 31% [26]. A study of 15 mechanically ventilated ICU patients with cirrhosis and AKI who were treated with continuous venovenous hemodialysis (CVVHD) described a median survival of 4 days and a 30-day survival of 0% [21].

One study of KRT in AKI and cirrhosis included 478 patients initiated on HD or continuous KRT for AKI [22]. Patients were stratified by cause of AKI and transplant listing status. The authors found a median transplant-free survival of 14 days regardless of cause of AKI among listed and non-listed subjects. Among non-listed subjects, 15% were alive at 6 months, and among patients admitted or transferred to the ICU, 12% were alive at 6 months. Another study included 266 patients with ATN or HRS-AKI who were ineligible for transplantation and found a median survival of 12.5 days in the cohort initiated on KRT as compared to only 2 days in those who did not receive KRT [24].

The largest study on this subject was done in Canada with 722 patients with cirrhosis who developed AKI, 626 of whom were admitted to ICU. The authors found that 68% of patients died and 26% had renal recovery by 12 months. Liver transplant status was the strongest predictor of kidney recovery [27]. Only one previous study looked at the critically ill cirrhotic population with AKI where 193 patients requiring KRT were noted to have 83% mortality at 28 days which increased to 92% by one year [23]. These outcomes were very similar to ours.

KRT is primarily used as a bridge to transplantation rather than to reverse high short-term mortality in this patient population. We observed a survival measurable in days from KRT initiation. This reinforces prior observations that outcomes are not only poor but also similar across AKI etiologies when patients are critically ill in the ICU with multiorgan failure.

We followed a cohort of 131 patients with decompensated cirrhosis, medical ICU admission, and AKI or acute on chronic kidney disease (AOCKD) requiring KRT initiation and confirmed the grave prognosis of this patient population with survival typically limited to short term. For example, we observed a median survival of 5 days from the start of KRT. When stratified by cause of AKI, we found no significant differences in survival between patients with HRS-AKI and ATN. Similar to previous reports [22], these data underscore that dialysis-related complications in this patient population are related to the severity of liver and multiorgan failure rather than the cause of AKI. Therefore, offering dialysis only to patients with “reversible” AKI – i.e., ATN – may not meaningfully improve outcomes and should be considered in the broader clinical context.

In unadjusted analysis, younger age, absence of coronary artery disease, HD as the initial KRT modality, absence of intravenous vasopressor use in the first 24 h of ICU admission, and lower CLIF-C ACLF score were all associated with improved survival. The apparent survival differences observed across KRT modalities should be interpreted cautiously, as continuous therapies were more frequently used in patients with greater hemodynamic instability, raising the likelihood of confounding by indication. Notably, initial KRT modality was not independently associated with survival in adjusted analyses, suggesting that the unadjusted differences primarily reflect underlying illness severity rather than a true modality-specific effect. These considerations are particularly important given that patients receiving continuous KRT may have experienced more severe or evolving critical illness prior to initiation—factors not fully captured in the available data. Accordingly, these findings should be interpreted as exploratory and hypothesis-generating, given residual confounding, the retrospective study design, and constraints of the multivariable modeling strategy.

Only CLIF-C ACLF score remained independently associated with survival in adjusted analysis, whereas MELDNa did not. Importantly, CLIF-C ACLF score incorporates multi-organ dysfunction and has been shown to better risk stratify patients with decompensated cirrhosis [28]. Taken together, these findings reinforce that outcomes in critically ill patients with cirrhosis and AKI are driven predominantly by the overall severity of ACLF and the extent of extrahepatic organ dysfunction, rather than kidney failure in isolation or the specific modality of KRT. AKI requiring dialysis in this setting appears to function primarily as a marker of advanced systemic illness. Mortality risk is determined largely by the number and severity of failing organ systems rather than liver-specific parameters alone. Accordingly, prognostic tools such as CLIF-C ACLF, which integrate dynamic and multiorgan dysfunction, may better capture risk in this population than traditional liver-focused scores. This association may also reflect unmeasured aspects of evolving critical illness not captured by static baseline assessments.

Transplant candidacy represents a critical contextual factor in the prognosis of critically ill patients with cirrhosis and AKI requiring KRT and the outcomes of patients receiving KRT as a bridge are reportedly better. For example, Wong et al. followed 102 liver transplant candidates receiving KRT and noted 15.6% mortality at 3-month and 30% mortality at 1 year post transplantation [26]. In our cohort, transplant listing status at ICU admission was not clearly associated with survival though there may be a trend toward improved survival at 6 months. This likely reflects the dynamic nature of transplant candidacy and its imperfect relationship with eventual transplantation. Though all but one patient in our study who were transplanted survived to 6 months, listing for transplantation was not predictive of undergoing organ transplantation. In fact, only 4 of the 21 patients listed were eventually transplanted. Conversely, 5 of the 9 patients who eventually received organ transplant were not listed at time of ICU admission. This discordance likely reflects both underlying biological severity of illness and differences in treatment intensity along with evolving goals of care during critical illness. Patients considered potential transplant candidates may be managed with a greater emphasis on aggressive supportive care as a bridge to transplantation, whereas non-listed patients may more frequently transition toward comfort-focused care as prognosis becomes clearer. Accordingly, transplant listing status should be interpreted as a dynamic clinical designation rather than a fixed prognostic variable, and the absence of an observed association should not be interpreted as evidence that transplant-related context is unimportant. Rather, listing status alone does not reliably risk-stratify patients with respect to survival or identify those most likely to benefit from KRT. These findings further highlight the importance of integrating prognostic assessment with early, ongoing discussions regarding goals of care.

In addition, baseline differences between groups, particularly by initial KRT modality and transplant listing status, likely reflect underlying differences in illness severity and clinical decision-making. Patients selected for continuous KRT are more likely to have greater hemodynamic instability, while transplant listing status reflects evolving assessments of candidacy and prognosis. These factors are important for contextualizing observed survival patterns but granular baseline comparisons by subgroup were not performed given the retrospective design, limited sample size, and risk of further reducing statistical power.

We also observed that treatment-limitation decisions were common in this cohort. The inpatient palliative medicine service was consulted in only one-quarter of cases, while 96% of patients who died were receiving hospice or “comfort measures only” care at the time of death. Only 3 patients in the entire cohort underwent CPR prior to death. These findings suggest that outcomes were shaped not only by disease severity but also by clinical decision-making regarding the appropriateness of ongoing life-sustaining therapies, including KRT. However, we do not have granular data around this decision to make definitive conclusions and mortality should be interpreted within the context of both illness severity and evolving goals of care. Interestingly, previous studies have shown that palliative care referral rarely occurs in non-listed patients with cirrhosis [29], although utilization in the United States does appear to be increasing [30] and a recent review does suggest a path forward [31]. As might be expected, the palliative care service in our study tended to be involved more frequently in subjects not listed for liver transplantation (30%) as compared to listed subjects (5%).

From a clinical perspective, these findings should not be interpreted as evidence of futility of KRT, but rather as providing prognostic and risk stratification context to support shared decision-making. In critically ill patients with cirrhosis and AKI, particularly those without immediate transplant potential, prognostic tools such as CLIF-C ACLF may help clinicians, patients, and families develop realistic expectations regarding short- and medium-term outcomes while aligning treatment decisions with individual goals of care.

Future studies are needed to refine prognostic assessment and decision-making in critically ill patients with cirrhosis and AKI requiring KRT. Prospective, multicenter investigations incorporating standardized criteria for KRT initiation and modality selection would help reduce selection bias and improve generalizability. In addition, structured causal approaches such as propensity-based analyses or time-dependent modeling may better account for evolving illness severity, treatment selection, and care-limitation decisions that cannot be addressed in retrospective cohorts. Integration of dynamic severity measures, including trajectories of organ failure, vasopressor requirements, lactate levels, and markers of systemic inflammation, may further improve prognostic frameworks beyond static baseline scores. Together, such approaches may help move the field toward more accurate, ICU-relevant prognostic tools to support goal-concordant care rather than definitive treatment algorithms.

This study must be considered within the context of some limitations. First, this was an observational design; we relied on medical records and the assessments of consultants to discern the cause of AKI. This does not allow for a standardized and reproducible approach to elicit the cause of AKI. It is therefore possible that some patients were miscategorized as ATN or HRS-AKI but since previous studies also did not find a difference based on etiology of AKI, we do not expect this to skew the results. Second, this study was done at a single tertiary transplant center and may limit generalizability due to differences in practice patterns. Third, the cohort included only patients in whom KRT was initiated, and the total number of ICU patients with cirrhosis and AKI who were not offered KRT could not be reliably determined. As a result, the study population represents a selected subgroup and observed survival estimates may be substantially influenced by unmeasured selection mechanisms. Accordingly, these findings should not be interpreted as representative of all critically ill patients with cirrhosis and AKI. Fourth, the multivariable modeling strategy was exploratory and based on univariable statistical significance, which may have excluded clinically relevant variables such as markers of respiratory or hemodynamic failure. This decision was made to reduce collinearity and model overfitting given limited sample size. In addition, proportional hazards assumptions were not formally tested, and analyses were conducted using a complete-case approach without formal assessment of missing data patterns. These factors limit confidence in identifying independent prognostic associations, and the reported hazard ratios should be interpreted as exploratory signals rather than definitive effect estimates. Additionally, given the limited number of outcome events and survivors at prespecified time points, we did not perform additional discrimination analyses as such analyses would likely yield statistically unstable and potentially misleading estimates in this modest-sized cohort. Fifth, we did not look at outcomes for patients who were not offered or who declined dialysis, and hence there is no “control” group for comparison. Sixth, adjustment for severity of illness was limited by the availability of static baseline measures. While MELD-Na and CLIF-C ACLF scores were calculated at ICU admission and laboratory data were collected at the time of KRT initiation, important dynamic aspects of critical illness were not captured. These include the timing of KRT initiation relative to ICU admission, vasopressor dose and intensity, and other granular hemodynamic data. As a result, we were unable to account for the trajectory of illness leading up to KRT initiation, which may confound observed associations between clinical variables and outcomes. Lastly, because the short-term mortality rate was so high and the size of the cohort was relatively small, we were unable to prove significant associations between mortality with several clinical markers.

## Conclusion

In this observational cohort of critically ill patients with cirrhosis and AKI requiring KRT, survival following KRT initiation was limited, with median survival measured in days. Mortality was primarily associated with overall severity of liver failure, as reflected by CLIF-C ACLF score, rather than AKI etiology or transplant listing status. Although unadjusted survival differed by initial KRT modality, this likely reflects confounding by illness severity rather than a modality-specific effect. These findings should be interpreted as descriptive and prognostic, not as evidence of futility or harm of KRT. Instead, they highlight the importance of severity-based risk stratification to inform shared, goal-concordant decision-making, particularly in patients without immediate transplant potential, and support early integration of palliative care when appropriate.

## Data Availability

The datasets generated and/or analysed during the current study are not publicly available due to patient confidentiality but are available from the corresponding author on reasonable request.

## Abbreviations

AKI: Acute kidney injury
ICU: Intensive care unit
HRS AKI: Hepatorenal-acute kidney injury
KRT: Kidney replacement therapy
CKD: Chronic kidney disease
CVVH: Continuous veno venous hemofiltration
HD: Hemodialysis
CVVHD: Continuous veno venous hemodialysis
CPR: Cardiopulmonary resuscitation

## References

[1] Murray CJ, Atkinson C, Bhalla K, Birbeck G, Burstein R, Chou D, et al. The state of US health, 1990–2010: burden of diseases, injuries, and risk factors. JAMA. 2013;310(6):591–608. 23842577 10.1001/jama.2013.13805 PMC5436627

[2] Muciño-Bermejo J, Carrillo-Esper R, Uribe M, Méndez-Sánchez N. Acute kidney injury in critically ill cirrhotic patients: a review. Ann Hepatol. 2012;11(3):301–10. 22481447

[3] Belcher JM, Garcia-Tsao G, Sanyal AJ, Bhogal H, Lim JK, Ansari N, et al. Association of AKI with mortality and complications in hospitalized patients with cirrhosis. Hepatology. 2013;57(2):753–62. 22454364 10.1002/hep.25735 PMC3390443

[4] Fagundes C, Barreto R, Guevara M, Garcia E, Solà E, Rodríguez E, et al. A modified acute kidney injury classification for diagnosis and risk stratification of impairment of kidney function in cirrhosis. J Hepatol. 2013;59(3):474–81. 23669284 10.1016/j.jhep.2013.04.036

[5] Piano S, Rosi S, Maresio G, Fasolato S, Cavallin M, Romano A, et al. Evaluation of the Acute Kidney Injury Network criteria in hospitalized patients with cirrhosis and ascites. J Hepatol. 2013;59(3):482–9. 23665185 10.1016/j.jhep.2013.03.039

[6] Desai AP, Knapp SM, Orman ES, Ghabril MS, Nephew LD, Anderson M, et al. Changing epidemiology and outcomes of acute kidney injury in hospitalized patients with cirrhosis - a US population-based study. J Hepatol. 2020;73(5):1092–9. 32387698 10.1016/j.jhep.2020.04.043 PMC7994029

[7] Garcia-Tsao G, Parikh CR, Viola A. Acute kidney injury in cirrhosis. Hepatology. 2008;48(6):2064–77. 19003880 10.1002/hep.22605

[8] Karvellas CJ, Durand F, Nadim MK. Acute Kidney Injury in Cirrhosis. Crit Care Clin. 2015;31(4):737–50. 26410141 10.1016/j.ccc.2015.06.009

[9] Davenport A, Sheikh MF, Lamb E, Agarwal B, Jalan R. Acute kidney injury in acute-on-chronic liver failure: where does hepatorenal syndrome fit? Kidney Int. 2017;92(5):1058–70. 28844314 10.1016/j.kint.2017.04.048

[10] Chaney A. A Review for the Practicing Clinician: Hepatorenal Syndrome, a Form of Acute Kidney Injury, in Patients with Cirrhosis. Clin Exp Gastroenterol. 2021;14:385–96. 34675586 10.2147/CEG.S323778 PMC8502008

[11] Angeli P, Garcia-Tsao G, Nadim MK, Parikh CR. News in pathophysiology, definition and classification of hepatorenal syndrome: A step beyond the International Club of Ascites (ICA) consensus document. J Hepatol. 2019;71(4):811–22. 31302175 10.1016/j.jhep.2019.07.002

[12] Wadei HM, Mai ML, Ahsan N, Gonwa TA. Hepatorenal syndrome: pathophysiology and management. Clin J Am Soc Nephrol. 2006;1(5):1066–79. 17699328 10.2215/CJN.01340406

[13] Ginès P, Solà E, Angeli P, Wong F, Nadim MK, Kamath PS. Hepatorenal syndrome. Nat Rev Dis Primers. 2018;4(1):23. 30213943 10.1038/s41572-018-0022-7

[14] Nadim MK, Garcia-Tsao G. Acute Kidney Injury in Patients with Cirrhosis. N Engl J Med. 2023;388(8):733–45. 36812435 10.1056/NEJMra2215289

[15] Gonwa TA, Wadei HM. The challenges of providing renal replacement therapy in decompensated liver cirrhosis. Blood Purif. 2012;33(1–3):144–8. 22269395 10.1159/000334149

[16] Belcher JM. Is there a role for dialysis in patients with hepatorenal syndrome who are not liver transplant candidates? Semin Dial. 2014;27(3):288–91. 24620917 10.1111/sdi.12224

[17] Capling RK, Bastani B. The clinical course of patients with type 1 hepatorenal syndrome maintained on hemodialysis. Ren Fail. 2004;26(5):563–8. 15526916 10.1081/jdi-200035988

[18] Nadkarni GN, Simoes PK, Patel A, Patel S, Yacoub R, Konstantinidis I, et al. National trends of acute kidney injury requiring dialysis in decompensated cirrhosis hospitalizations in the United States. Hepatol Int. 2016;10(3):525–31. 26825548 10.1007/s12072-016-9706-9

[19] Keller F, Heinze H, Jochimsen F, Passfall J, Schuppan D, Büttner P. Risk factors and outcome of 107 patients with decompensated liver disease and acute renal failure (including 26 patients with hepatorenal syndrome): the role of hemodialysis. Ren Fail. 1995;17(2):135–46. 7644764 10.3109/08860229509026250

[20] Wilkinson SP, Weston MJ, Parsons V, Williams R. Dialysis in the treatment of renal failure in patients with liver disease. Clin Nephrol. 1977;8(1):287–92. 884909

[21] Witzke O, Baumann M, Patschan D, Patschan S, Mitchell A, Treichel U, et al. Which patients benefit from hemodialysis therapy in hepatorenal syndrome? J Gastroenterol Hepatol. 2004;19(12):1369–73. 15610310 10.1111/j.1440-1746.2004.03471.x

[22] Allegretti AS, Parada XV, Eneanya ND, Gilligan H, Xu D, Zhao S, et al. Prognosis of Patients with Cirrhosis and AKI Who Initiate RRT. Clin J Am Soc Nephrol. 2018;13(1):16–25. 29122911 10.2215/CJN.03610417 PMC5753306

[23] Staufer K, Roedl K, Kivaranovic D, Drolz A, Horvatits T, Rasoul-Rockenschaub S, et al. Renal replacement therapy in critically ill liver cirrhotic patients-outcome and clinical implications. Liver Int. 2017;37(6):843–50. 28211257 10.1111/liv.13389

[24] Diaz PM, Saly DL, Horick N, Petrosyan R, Gitto Z, Indriolo T, et al. Prognosis of Transplant-Ineligible Patients with Cirrhosis and Acute Kidney Injury Who Initiate Renal Replacement Therapy. Dig Dis Sci. 2024;69(10):3710–20. 39215868 10.1007/s10620-024-08623-2 PMC11647743

[25] Arroyo V, Moreau R, Jalan R. Acute-on-Chronic Liver Failure. N Engl J Med. 2020;382(22):2137–45. 32459924 10.1056/NEJMra1914900

[26] Wong LP, Blackley MP, Andreoni KA, Chin H, Falk RJ, Klemmer PJ. Survival of liver transplant candidates with acute renal failure receiving renal replacement therapy. Kidney Int. 2005;68(1):362–70. 15954928 10.1111/j.1523-1755.2005.00408.x

[27] Wang PL, Silver SA, Djerboua M, Thanabalasingam S, Zarnke S, Flemming JA. Recovery From Dialysis-Treated Acute Kidney Injury in Patients With Cirrhosis: A Population-Based Study. Am J Kidney Dis. 2022;80(1):55–e641. 34808296 10.1053/j.ajkd.2021.09.025

[28] Jalan R, Saliba F, Pavesi M, Amoros A, Moreau R, Ginès P, et al. Development and validation of a prognostic score to predict mortality in patients with acute-on-chronic liver failure. J Hepatol. 2014;61(5):1038–47. 24950482 10.1016/j.jhep.2014.06.012

[29] Poonja Z, Brisebois A, van Zanten SV, Tandon P, Meeberg G, Karvellas CJ. Patients with cirrhosis and denied liver transplants rarely receive adequate palliative care or appropriate management. Clin Gastroenterol Hepatol. 2014;12(4):692–8. 23978345 10.1016/j.cgh.2013.08.027

[30] Rush B, Walley KR, Celi LA, Rajoriya N, Brahmania M. Palliative care access for hospitalized patients with end-stage liver disease across the United States. Hepatology. 2017;66(5):1585–91. 28660622 10.1002/hep.29297

[31] Bansal AD, Patel AA. Dialysis initiation for patients with decompensated cirrhosis when liver transplant is unlikely. Curr Opin Nephrol Hypertens. 2024;33(2):212–9. 38038622 10.1097/MNH.0000000000000959

